# High Resolution ^31^P Field Cycling NMR Reveals Unsuspected Features of Enzyme-Substrate-Cofactor Dynamics

**DOI:** 10.3389/fmolb.2022.865519

**Published:** 2022-03-31

**Authors:** Mary F. Roberts, Lizbeth Hedstrom

**Affiliations:** ^1^ Department of Chemistry, Boston College, Chestnut Hill, MA, United States; ^2^ Departments of Biology and Chemistry, Brandeis University, Waltham, MA, United States

**Keywords:** GMP reductase, field cycling, enzyme dynamics, ^31^P NMR, relaxometry, ligand dynamics, dipolar relaxation

## Abstract

The dynamic interactions of enzymes and substrates underpins catalysis, yet few techniques can interrogate the dynamics of protein-bound ligands. Here we describe the use of field cycling NMR relaxometry to measure the dynamics of enzyme-bound substrates and cofactors in catalytically competent complexes of GMP reductase. These studies reveal new binding modes unanticipated by x-ray crystal structures and reaction-specific dynamic networks. Importantly, this work demonstrates that distal interactions not usually considered part of the reaction coordinate can play an active role in catalysis. The commercialization of shuttling apparatus will make field cycling relaxometry more accessible and expand its use to additional nuclei, promising more intriguing findings to come.

## Introduction

The extraordinary power of enzyme catalysis relies on the dynamic alignment of substrates and active site residues. While X-ray crystal structures provide invaluable insights into enzyme-substrate interactions, such structures are typically static views of chemically inert complexes. Tremendous progress has been made using NMR methods to measure protein dynamics, but such studies have largely focused on changes in protein conformation and identification of ligand binding sites, again with typically chemically inert complexes (Akke, 2012; Palmer, 2015; Bax and Clore, 2019; Strotz et al., 2020). These experiments have limited utility for large multimeric proteins. Paramagnetic relaxation can be used to measure ligand binding and infer conformation if the enzyme contains a suitable spin center (Li et al., 2012; Softley et al., 2020). Substrate dynamics can also be addressed computationally, but proper benchmarking is difficult given the few experimental methods available to validate or corroborate such studies (Chakravorty and Merz, 2015). Here we describe the use of high resolution ^31^P field cycling NMR relaxometry to investigate the dynamics of enzyme-bound substrates and cofactors. These experiments provide a unique ligand-centric view of protein-ligand interactions. We describe work in the model system of GMP reductase (GMPR) (Rosenberg et al., 2016; Rosenberg et al., 2018; Rosenberg et al., 2020), where these experiments reveal a novel binding mode unanticipated by crystal structures as well as reaction-specific dynamic networks. Importantly, this work demonstrates that substrate/cofactor phosphates and other interactions not usually considered part of the reaction coordinate can be active participants in catalysis rather than passive bystanders as often assumed.

### The Method

**FIGURE 1:**
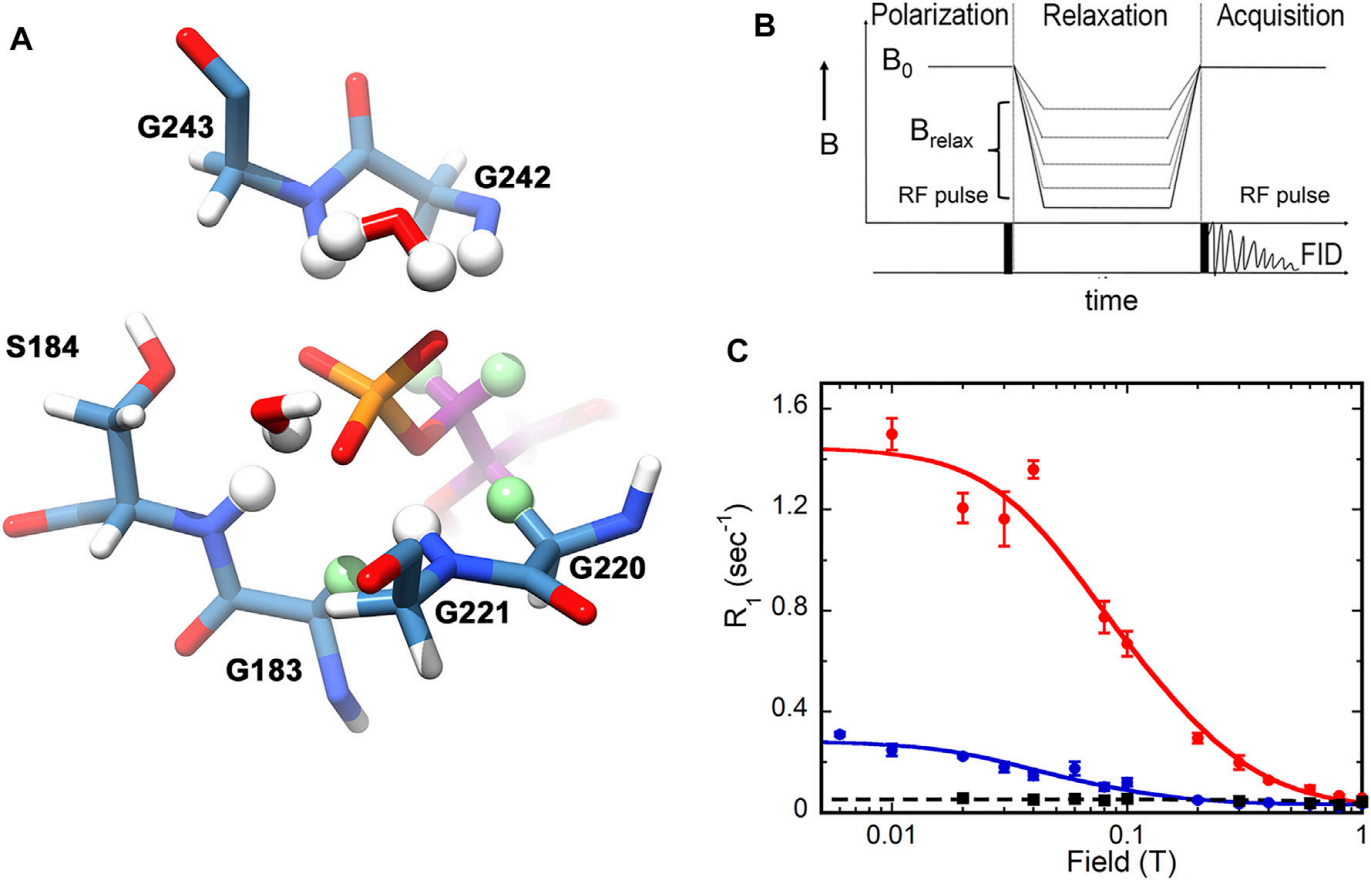
^31^P relaxation as measured by high resolution relaxometry. **(A)** Structure of the IMP monophosphate binding site in the E•IMP•NADP^+^ complex of GMPR (pdb 2c6q). Residues within 4 Å of the IMP ^31^P are shown. Protein carbon atoms are colored steel blue, IMP carbon atoms are dark magenta, potential ^1^H relaxers are shown as balls, nonexchangeable ^1^H relaxers are light green. This figure was produced with UCSF Chimera (Pettersen et al., 2004). **(B)** Design of the field cycling experiment. **(C)** Examples of field cycling data for substrate monophosphates in ternary complexes with GMPR and cofactor (Rosenberg et al., 2016). The relaxation of IMP is shown in red and GMP in blue. The black trace shows the relaxation of IMP in the absence of enzyme. Note that the CSA component of relaxation is not observed since the field axis is only shown to 1 T and R_CSA_ is not observed until the magnetic field is greater than 5 T. **(B,C)** were reprinted with permission from (Rosenberg et al., 2018). Copyright 2020 American Chemical Society.

The power of subtesla high-resolution field-cycling NMR relaxometry to interrogate the dynamics of enzyme-bound substrates is only beginning to be appreciated (Roberts and Redfield, 2004; Pu et al., 2009; Pu et al., 2010; Redfield, 2012; Gradziel et al., 2014; Wei et al., 2015; Roberts et al., 2021; Wang et al., 2021). Dipolar relaxation results from the interaction of a nucleus of interest, e.g., ^31^P, with nearby dipoles, usually ^1^H. An example of potential ^31^P—^1^ H dipolar interactions for an enzyme-bound substrate is shown in Figure 1A. Thus dipolar relaxation reflects the structure of the binding site and the mobility of substrate. However, chemical shift anisotropy (CSA) is the mechanism that dominates the spin-lattice/longitudinal rate (R_1_) of ^31^P in the high magnetic fields of current spectrometers. Dipolar relaxation can be observed at lower magnetic fields where CSA relaxation is minimal, but differences in the chemical shifts of multiple ^31^P in a sample are lost. In a high resolution field cycling relaxometry experiment, samples are excited at high magnetic field, shuttled up the bore of the magnet to low field for relaxation, then shuttled back to high field to detect the residual magnetization (Figure 1B) (Roberts and Redfield, 2004; Redfield, 2012). This type of field cycling preserves chemical shift information, allowing the magnetic field dependence of R_1_ to be measured simultaneously for different ^31^P nuclei. Importantly, the enzyme-bound substrate must be in fast exchange with free substrate, making the observed R_1_ the weighted average of the very small R_1_ of the free substrate and the much larger R_1_ of the enzyme-bound substrate (which is a function of the correlation time of the enzyme•substrate complex) (Pu et al., 2009). Under these conditions, the experiments characterize the ground state Michaelis complex.

In a typical high resolution relaxometry experiment (e.g., Figure 1C), R_1_ is measured at varying magnetic fields ranging from 0.003 to 11.7 T, yielding two dipolar parameters: 1) R_D_(0), the maximum dipolar relaxation rate at zero field; and 2) τ_D_, the molecular dipolar correlation time (Roberts and Redfield, 2004; Redfield, 2012). The overall R_1_ at any magnetic field is then the sum of dipolar and CSA R_1_ values. Importantly, the ratio τ_D_/R_D_(0) is related to the sixth power of the averaged effective distance (r_eff_) between the ^31^P nuclei and the ^1^H relaxers. These relationships are described in equations 1-3.

$$R_{1}=\left(\frac{R_{D}\left(0\right)}{2\tau_{D}}\right)\left\{0.1J\left(\tau_{D},\omega_{H}–\omega_{P}\right)+0.3J\left(\tau_{D},\omega_{P}\right)+0.6J\left(\tau_{D},\omega_{H}+\omega_{P}\right)\right\}+k_{D}\;+\;k_{CSA}\omega_{P}^{2}\;\;$$ (1)

$$r_{eff}^{6}=\frac{{\left({µ}_{0}/4\pi \right)}^{2}{\left(h/2\pi \right)}^{2}\gamma_{P}^{2}\gamma_{H}^{2}\tau_{D}}{R_{D}\left(0\right)}$$ (2)

where

$$J\left(\tau_{D},\omega \right)=\frac{2\;\tau_{D}}{\left(1+\omega^{2}\tau_{D}^{2}\right)}$$ (3)

and k_D_ is the R_D_(0) for very fast dipolar relaxation of the ^31^P where τ_D_ < 0.5 ns (Roberts et al., 2021). The R_CSA_ term, k_CSA_ ω_P_^2^, assumes that ω^2^τ_CSA_^2^ < 1; the square law increase in R_1_ is not detected until the relaxation field, B_relax_, is >5 T. In the expression for r_eff_, µ_0_ is the magnetic permeability in a vacuum, h is Planck’s constant, and the two *γ* are gyromagnetic ratios for ^31^P and ^1^H.

For the purposes of this review, discussion will be limited to τ_D_ and τ_D_/R_D0_. If the bound substrate is relatively rigid, then the value of τ_D_ will be comparable to the overall rotation of the enzyme complex, but if the substrate is mobile, the values of R_D_(0) and τ_D_ can be reduced. As noted above, the sixth root of τ_D_/R_D0_ is related to the averaged effective distance (r_eff_) between the ^31^P nucleus and the ^1^H relaxers, so that smaller values of τ_D_/R_D0_ indicate that the relaxers are closer and/or more relaxers are present. The ^1^H relaxers can be intramolecular (e.g., the 5′-^1^H nuclei of IMP in Figure 1A), in which case their contribution to relaxation is determined by the conformation of the substrate, or intermolecular (e.g., αC^1^H_2_ nuclei of Gly183 and Gly220 in Figure 1A), where their contribution is determined by the structure of the binding site. The ^1^H relaxers can also be exchangeable protons or even water. Thus, high resolution relaxometry experiments probe critical structural and dynamic features of substrate binding sites.

### The System

Given the widespread prevalence of phosphorylated metabolites in critical biochemical pathways, there are surprisingly few studies that utilize ^31^P NMR relaxation, and specifically spin-lattice/longitudinal relaxation, to investigate enzyme-substrate dynamics. GMPR is a particularly attractive system for such studies because the reaction involves four ^31^P nuclei, one on the GMP/IMP substrate and three on the NADP^+^ cofactor. Moreover, the cofactor undergoes a conformational change during the catalytic cycle (Patton et al., 2011), suggesting that relaxation studies will provide new insights into the reaction. As described below, two dead-end yet catalytically competent complexes are available, mimicking each step of the catalytic cycle. GMPR is an (*α*/*β*)_8_ “TIM” barrel, the most common enzyme fold, with the standard phosphate “gripper” loop conserved throughout the TIM superfamily (Wilmanns et al., 1991; Nagano et al., 2002). Therefore investigation of GMPR is likely to provide insight into many enzyme reactions.

**FIGURE 2:**
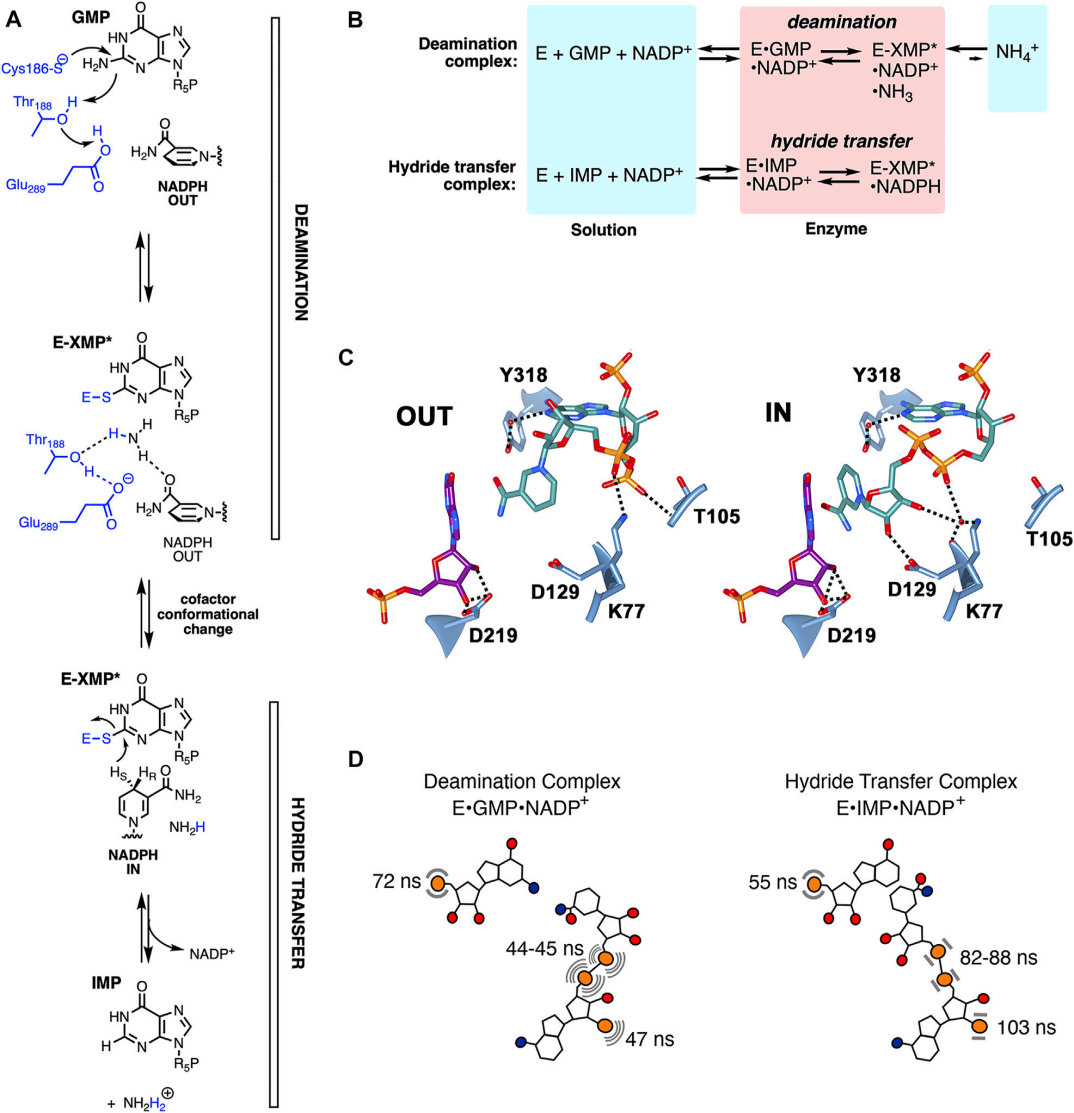
The dynamics of enzyme-bound substrate and cofactors in GMPR. **(A)** The GMPR reaction. **(B)** Partial reactions catalyzed by GMPR. **(C)** Cofactor conformations observed in E•IMP•NADP^+^ crystal structure (pdb 2c6q). IMP carbon atoms are shown in dark magenta, NADP^+^ in dark cyan, hydrogen bonds are depicted with dashed lines. This figure was produced with UCSF Chimera (Pettersen et al., 2004). **(D)** Cartoon showing the values of τ_D_ in the deamination and hydride transfer complexes. The cofactor is more dynamic than the substrate in the deamination complex while the substrate is more dynamic than the cofactor in the hydride transfer complex. **(A,B)** were reprinted with permission from (Rosenberg et al., 2020). Copyright 2020 American Chemical Society.

GMPR catalyzes the reduction of GMP to IMP and ammonia with concomitant oxidation of NADPH. The reaction proceeds via two steps (Figure 2A): 1) Deamination of GMP via attack of the catalytic Cys to produce the thioimidate intermediate E-XMP* and ammonia followed by 2) hydride transfer from NADPH to E-XMP* producing IMP. Two catalytically active, yet dead end, complexes provide windows into each step (Figure 2B): 1) The E•GMP•NADP^+^ complex can undergo deamination to form E-XMP*•NH_3_•NADP^+^ but cannot proceed to products in the absence of NADPH; 2) hydride transfer can occur in the E•IMP•NADP^+^ complex to form E-XMP*•NADPH, but the reaction cannot proceed to GMP in the absence of ammonia. The cofactor is present throughout the catalytic cycle (Patton et al., 2011), and must adopt different conformations in each step. Two cofactor conformations are found in the x-ray crystal structure of the inactive E•IMP•NADPH complex (Figure 2C) (Patton et al., 2011). The nicotinamide is far from the substrate in the “OUT” conformation, as would be expected during the deamination reaction. In contrast, the nicotinamide is stacked with the substrate as required for hydride transfer in the “IN” conformation. The two ^31^P nuclei of the cofactor diphosphate occupy different sites in the IN and OUT conformations, while the substrate and cofactor monophosphates appear to occupy the same site in both complexes. If the crystal structures accurately reflect catalytically competent structures, then the cofactor diphosphates are expected to be mobile while the substrate and cofactor monophosphates are constrained.

### Field Cycling Reveals a Novel Binding Site and Reaction-specific Dynamics

The values of τ_D_ reveal surprising differences in the mobilities of enzyme bound substrates and cofactors (Rosenberg et al., 2016; 2018). As noted above, if the substrate/cofactor is rigidly bound, then the value of τ_D_ should approximate the rotational correlation time for the enzyme. GMPR is a homotetramer of 37 kDa subunits. For a spherical protein of this size, the correlation time can be estimated to be ∼70 ns using Stokes law. However, GMPR is disk-like, with multiple rotational axes, each with a distinct correlation time, corresponding to a larger observed τ_D_ (Ortega and Torre, 2003). The observed values of τ_D_ for ^31^P nuclei bound to wild-type GMPR complexes are as high as 103 ns (Rosenberg et al., 2016). Mutant enzyme complexes, e.g., K77A and D129A, can display ^31^P τ_D_ values of 160–195 ns (Rosenberg et al., 2020), which may be the actual correlation time for the protein. The τ_D_ values were much less than 160 ns in most cases, suggesting that the enzyme-bound substrate and cofactor are mobile. Importantly, if the substrate and cofactor were both rigidly bound to the enzyme, the values of τ_D_ for all for ^31^P nuclei would be the same. As described in more detail below, the values of τ_D_ vary for different nuclei in different complexes, such that distinct dynamic signatures are observed for each partial reaction.

The values of τ_D_ for the ^31^P nuclei of GMP and IMP in their respective binary enzyme complexes were both 43 ns (±4 and ±6 ns, respectively), indicating that the monophosphates are mobile on the enzyme. The values of τ_D_/R_D_(0) for enzyme bound GMP and IMP are also very similar (1.9–2.0 × 10^−8^ s^2^ for τ_D_/R_D0_), indicating that the binding sites are essentially the same, as found in the crystal structures.

In contrast to the binary substrate complexes, the ternary enzyme-substrate-cofactor complexes display distinct dynamic profiles (Figures 1C, 2D) (Rosenberg et al., 2016; 2018). The dynamic properties of IMP are very similar in the absence and presence of NADP^+^. The cofactor increased the value of τ_D_ for GMP, suggesting that GMP is more rigidly bound than IMP in the cofactor complex. More importantly, a 10-fold increase is observed in the value of τ_D_/R_D_(0) for GMP, which indicates that the cofactor induces a new binding mode for GMP where the substrate monophosphate is farther from ^1^H relaxers than in the IMP•cofactor complex. This difference in substrate monophosphate binding modes was further substantiated when relaxation was measured in the presence of D_2_O, which increases τ_D_/R_D_(0) for the IMP ^31^P by 2.5-fold but has no effect on the relaxation of GMP ^31^P. Thus exchangeable ^1^H relaxers are present near the IMP monophosphate that are absent in the GMP binding site. These observations reveal a novel binding mode for GMP unanticipated by the crystal structures. The reaction-specific interactions induced by the cofactor indicate that the monophosphate does not simply tether the substrate to the active site but instead participates in the catalytic cycle. Since the substrate monophosphate binds in the conserved phosphate gripper motif found throughout the TIM superfamily, similar reaction-specific interactions may well occur in many other enzyme reactions.

The cofactor displays different dynamic properties in the deamination and hydride transfer complexes (Figure 2D). All three cofactor ^31^P nuclei are more mobile than the substrate monophosphate in the deamination complex, with values of τ_D_ ranging from 44 to 47 ns versus 72 ns for GMP. In contrast, the cofactor is more rigid than the substrate in the hydride transfer complex, with values of τ_D_ ranging from 82 to 103 ns versus 55 ns for IMP. While the differences in the dynamic behavior of the diphosphate ^31^P nuclei were anticipated from the crystal structures, the cofactor monophosphate was expected to exhibit similar dynamic features in both complexes. Thus, like the substrate monophosphate, the cofactor monophosphate is also intimately coupled to the catalytic cycle.

Although the crystal structure suggested that the cofactor should occupy different sites in the deamination and hydride transfer complexes, the values of τ_D_/R_D_(0) for the diphosphate ^31^P nuclei were similar, with overlapping errors (e.g., (9.2 ± 2.4) × 10^−8^ s^2^ and (6.6 ± 1.2) × 10^−8^ s^2^ for the deamination and hydride transfer complex, respectively). Close inspection of the crystal structures revealed similar numbers of ^1^H relaxers in the vicinity of the cofactor diphosphate ^31^P nuclei in the OUT and IN conformations (14 and 15, respectively, within 4 Å), which can explain the similar values of τ_D_/R_D_(0). However, fewer exchangeable ^1^H relaxers are observed in the OUT conformation (8) than in the IN conformation (13), suggesting that D_2_O should have a larger effect on the dynamic properties of the hydride transfer complex than the deamination complex. Indeed, the presence of D_2_O increased the values of τ_D_/R_D_(0) for the diphosphate ^31^P nuclei by a factor of 2 in the hydride transfer complex versus 1.5 in the GMP complex, as expected if the cofactor has different binding modes as suggested by the crystal structures.

### Distinct Enzyme•Substrate•Cofactor Interactions Modulate Reaction-Specific Dynamics

The field cycling experiments indicate that distinct dynamic behavior is associated with each step in the catalytic cycle. Mutagenesis and substrate analogs revealed that this behavior derives from specific enzyme•substrate•cofactor interactions. For example, the substrate 2′-OH forms a hydrogen bond with Asp219 in the crystal structures (Figure 2C). The removal of this interaction by creating an enzyme-cofactor complex with dIMP reduced the rate of hydride transfer by a factor of 6.7 (Rosenberg et al., 2018). The dynamics of this complex were dysregulated, with values of τ_D_ and τ_D_/R_D_(0) very similar to those of the native deamination complex. Thus the 2′-OH of IMP differentiates the two dynamic states. Further insight into the nature of the reaction-specific dynamics were revealed by substituting Asp219 with Ala. This mutation reduced the rate of deamination by 50-fold and caused widespread changes in dynamics (Rosenberg et al., 2018). Perhaps counterintuitively, the loss of the Asp219 interaction caused both substrate and cofactor to be constrained relative to the native deamination complex. Moreover, the substrate binding mode was not congruent with deamination but instead resembled that of the native hydride transfer complex. The D219A substitution had little effect on the rate of hydride transfer and dynamics of the E•IMP•cofactor complex. Thus Asp219 is part of a deamination-specific dynamic network.

Further exploration of the roles of active site residues revealed complex networks of dynamic interactions. Asp129 forms a hydrogen bond to the cofactor in the IN conformation in the crystal structure (Figure 2C), suggesting that it holds the cofactor in place for hydride transfer. This interaction is absent in the OUT conformation. Substitution of Asp129 with Ala reduces the rates of both deamination and hydride transfer by factors of 100 (Rosenberg et al., 2020). The dynamic profiles of the deamination and hydride transfer complexes were indistinguishable in the D129A variant, and both substrate and cofactor were constrained when bound to the enzyme, with values of τ_D_ ranging from 100 to 160 ns for the cofactor ^31^P nuclei. Thus Asp129 is a critical link in the dynamic networks of both reactions.

Lys77 makes hydrogen bonds to the cofactor, either direct or indirect via water, in both the OUT and IN conformations in the crystal structure. Substitution of Lys77 with Ala reduced the rate of deamination and hydride transfer by factors of 100 and 30, respectively (Rosenberg et al., 2020). This substitution had dramatic, yet reaction-specific, effects on the dynamics of both complexes. The dynamics of GMP and cofactor most closely resembled a native hydride transfer complex, and thus are not compatible with deamination, while IMP and cofactor were both constrained when bound to the K77A variant. These observations suggest that Lys77 also participates in the dynamic networks of both reactions. In contrast, Thr105, Tyr318 and Asp219 participate in the deamination-specific network but are not involved in hydride transfer. These findings provide a framework to identify correlated motions required for catalysis in the two steps of the GMPR reaction.

### Future Perspectives

While the field cycling experiments described above only begin to map the reaction-specific dynamic networks of GMPR, some themes have already emerged. First, critical dynamic interactions extend far beyond the sites of chemical transformation to seemingly inert moieties like the substrate monophosphate. Second, field cycling can reveal catalytically competent binding modes that elude crystal structures. Lastly, seemingly small structural perturbations of the substrate or enzyme such as the substitution of H for OH, can cause widespread and unpredictable changes in the dynamic behavior of substrates and cofactors. We have only just scratched the surface of the potential of high resolution ^31^P relaxometry given the prevalence of phosphorylated substrates and cofactors.

Until recently, shuttler systems for high resolution solution field cycling have been home-built (Ajoy et al., 2019; Charlier et al., 2013; Chou et al., 2012; Redfield, 2012; Roberts et al., 2004). The method will become more accessible with the availability of commercial add-on shuttling systems compatible with cryo-probes from Field Cycling Technology Ltd., Taiwan (Chou et al., 2012). Such systems will provide enhanced sensitivity and extend the methodology to other ligand nuclei (e.g., ^1^H, or specifically enriched ^13^C or ^15^N). We believe high resolution field cycling relaxometry is poised to reveal many more unsuspected features of the structure and dynamics of protein-ligand binding.
